# Exploring entropy measures in polymer graphs using logarithmic regression model

**DOI:** 10.1038/s41598-025-18050-6

**Published:** 2025-10-03

**Authors:** Muhammad Irfan, Jihad Younis, Alaa Altassan, Wafa F. Alfwzan, Nabeela Bashir, Nof T. Alharbi

**Affiliations:** 1 Department of Mathematics, University of Okara, Okara, Pakistan; 2 Department of Mathematics, Aden University, Khormaksar, Yemen; 3 Department of Mathematics, Faculty of Science, King Abdulaziz University, Jeddah, Saudi Arabia; 4 Department of Mathematical Sciences, College of Science, Princess Nourah Bint Abdulrahman University, Riyadh, Saudi Arabia; 5 Mathematics Department, University College in Al-Darb, Jazan University, Jazan, Saudi Arabia

**Keywords:** Regression model, Graph entropies, Polyester and polycarbonate, Vertex degree, Chemistry, Materials science, Mathematics and computing

## Abstract

Graph Entropies, calculated from indices, quantify the structural information of chemical linkages and graphs using Shannon’s Entropy notion. This study investigates the chemical structures of polyester and polycarbonate polymers. Polyesters are synthetic polymers consisting of repeating chemical units linked by covalent bonds formed through ester groups. They are versatile materials found in a wide range of everyday products. In contrast, polycarbonates are thermoplastic polymers characterized by carbonate groups. Renowned for their strength and toughness, polycarbonates are widely used in engineering applications. In this study, we compute the Zagreb indices, redefined Zagreb indices, atom-bond connectivity index, and geometric-arithmetic index for polymer structures using edge partitioning based on degrees. Furthermore, we calculate Entropy measures for these structures using Shannon’s Entropy notion. Through numerical computations, we compare the topological indices with their corresponding Entropy measures. Finally, we employ a regression model to analyze the relationship between these topological indices and Entropy metrics.

## Introduction

To model and analyze chemical networks, graph theory plays a vital role. Its application in chemistry aids in determining various features, including chemical activity, biological activity, thermodynamic properties, and physicochemical characteristics. Certain network invariants, known as topological indices, are used to characterize these features. Topological indices are numerical values that represent the topology of molecular structures in mathematical chemistry, as stated in reference^1^. They are widely applied in computational chemistry, chemoinformatics, and QSPR/QSAR studies to predict molecular properties such as reactivity, solubility, and biological activity based on structural features, as highlighted in the articles^2,3^.

Consider the graph $G = (V, E)$, in which the collection of vertices is represented by *V* and the set of edges is denoted by *E*. The degree of a vertex *m*, represented by $\nu _m$, is determined by the number of edges that are attached to it. The study and modeling of chemical compound structures is the main goal of chemical graph theory. Here, atoms serve as vertices and chemical bonds as edges in graphs that depict chemical compounds. A mathematical analysis of molecular structures using theoretical, computational, and graphical methods is presented in the references^4,5^. Gutman introduced the general form for degree-based topological indices:

$$\begin{aligned} TI(G)=\sum _{gh\in E(G)} f(\nu _g,\nu _h). \end{aligned}$$

Here, *TI*(*G*) denotes the topological index of the graph *G*. The term *gh* represents an edge connecting the vertices *g* and *h*, and *f* is a function that depends on the degrees of these vertices.

The Wiener index^6^, is the first topological index which was used to calculate the boiling points of alkanes, was introduced in 1947 by the renowned scientist Harold Wiener, who also pioneered the concept of topological indices. Among the earliest molecular descriptors in this field, the Zagreb indices were introduced by Gutman and Trinajstić^7,8^, who further observed in^9^ the influence of molecular structure on the overall energy of $\pi$-electrons. Amna Amer, M. Irfan, and Hamood ul Rehman calculated the edge versions of degree-based topological indices for boron triangular nanotubes in^10^, with related work also conducted by Amna Amer and collaborators in^11^. Additional, the degree-based topological indices which are widely used, these include the atom-bond connectivity index^12^, the redefined Zagreb indices^13^, and the geometric-arithmetic index^14^, which are frequently applied in modeling structure-property and structure-activity correlations.

Topological indices are mathematical descriptors used in chemistry and related fields to represent molecular structures. The degree of disorder or unpredictability in the distribution of specific structural elements is referred to as Entropy. Articles^15,16^ highlight several advantages of utilizing topological indices in molecular structures to compute Entropy. In his influential work^17^, Shannon introduced the concept of Entropy, describing it as “The entropy of a probability distribution is a measure of the unpredictability of information content or the uncertainty of a system.” Subsequently, Entropy has been employed to quantify the structural information of graphs and chemical networks. Rashevsky proposed the first concept of graph Entropy in 1955, using classifications of vertex orbits^18^. More recently, studies^19,20^ have demonstrated the extensive application of graph Entropies across various domains, including biology, ecology, sociology, and chemistry. While the degree power plays a critical role in network analysis, other graph types are also utilized for measuring Entropy. In their 2014 study, Chen et al.^21^ introduced a novel characterization of Entropy, presenting the concept of the Entropy of edge partitioned graphs.

1 $$\begin{aligned} ENT=-\sum _{i=1}^{r}U_i\frac{f(g_ih_i)}{TI}log\frac{f(g_ih_i)}{TI} =log(TI)-\frac{1}{TI}\sum _{i=1}^{r}U_ilogf(g_ih_i)^{f(g_ih_i)}. \end{aligned}$$

In this context, $TI = \sum _{i=1}^{r} U_i f(g_i h_i)$ represents the topological index, where $U_i$ is the frequency, *r* is the number of edges, and $f(g_i h_i)$ is the weight of the edge $g_i h_i$. Degree-based Entropies are valuable tools for analyzing the structure and behavior of complex networks. They provide insights into network robustness, community structure, and information propagation. Using a logarithmic regression model, Guofeng et al.^22^ reported the topological indices and Entropy metrics of the beryllonitrene network. In 2024, Ovais et al.^23^ calculated Entropy measures of dendrimers using degree-based indices. The topological Entropies of single-walled carbon nanotubes were computed by Raja and Anuradha in^24^. The results on degree-based graph Entropy in structure–property modeling, Zagreb connection indices in structure property modelling, On ve-degree irregularity index of graphs and its applications as molecular descriptor, Role of GA, AG and R in structure-property modelling and On exponential geometric-arithmetic index of graphs are published in^25–29^ respectively.

## Aim and methodology

This article aims to derive structural and informational properties of polymer graphs by employing information derived from degree-based topological indices, including the first Zagreb index, second Zagreb index, first redefined Zagreb index, second redefined Zagreb index, third redefined Zagreb index, atom bond connectivity index, and geometric arithmetic index, in order to calculate the degree-based Entropies of these structures.

The methodology of this article is structured as follows: Section “Degree-based topological indices” presents the definitions of degree-based topological indices of graphs. In Section “Degree-based entropies”, the concept of degree-based Entropy for graphs is introduced. Section “Polyester” provides the calculated results of degree-based Entropies and topological indices for polyester, supplemented with graphical representations and structural analysis. Section “Polycarbonate” focuses on the structure of polycarbonate, also presenting the corresponding degree-based Entropies and topological indices, along with visual illustrations. Section “Logarithmic model” explores the logarithmic model applied to both chemical graphs. In Section “Comparison and Discussion”, a comparative discussion of the results obtained for polyester and polycarbonate is provided. Finally, Section “Conclusion” concludes the article by summarizing the key findings.

## Degree-based topological indices

Gutman and Trinajstić introduced the first Zagreb index in 1972^8^, while Gutman and Das defined the second Zagreb index in 2004^7^.

2 $$\begin{aligned} M_1(G)= & \sum _{gh\in E(G)}(\nu _g+ \nu _h). \end{aligned}$$

3 $$\begin{aligned} M_2(G)= & \sum _{gh\in E(G)}(\nu _g\times \nu _h). \end{aligned}$$

Ranjini et al.^13^ in 2013 defined the first, second, and third redefined Zagreb indices for a graph as follows.

4 $$\begin{aligned} ReZG_1(G)= & \sum _{gh\in E(G)}{\frac{\nu _g+ \nu _h}{\nu _g\times \nu _h}}. \end{aligned}$$

5 $$\begin{aligned} ReZG_2(G)= & \sum _{gh\in E(G)}{\frac{\nu _g\times \nu _h}{\nu _g+ \nu _h}}. \end{aligned}$$

6 $$\begin{aligned} ReZG_3(G)= & \sum _{gh\in E(G)}(\nu _g\times \nu _h)(\nu _g+ \nu _h). \end{aligned}$$

Estrada et al. proposed the atom bond connectivity index in 1998^12^, which was stated as follows:

7 $$\begin{aligned} ABC(G)=\sum _{gh\in E(G)}\sqrt{\frac{\nu _g+\nu _h-2}{\nu _g \times \nu _h}}. \end{aligned}$$

Vukićevič and Furtula^14^ first introduced the geometric arithmetic index in 2009. The definition is as follows:

8 $$\begin{aligned} GA(G)=\sum _{gh\in E(G)}{\frac{2\sqrt{\nu _g \times \nu _h}}{\nu _g+\nu _h}}. \end{aligned}$$

## Degree-based entropies

- *First Zagreb Entropy:* If $TI=\sum _{gh\in E(G)}(\nu _g+ \nu _h)=M_1(G)$, then Eq. 1 reduced in the following form and is known as the first Zagreb Entropy.
  9 $$\begin{aligned} ENT_{M_1(G)}=log[M_1(G)]-\frac{1}{M_1(G)}\sum _{i=1}^{r}U_ilog[\nu _g+ \nu _h]^{[\nu _g+ \nu _h]}. \end{aligned}$$
- *Second Zagreb Entropy:* If $TI=\sum _{gh\in E(G)}(\nu _g\times \nu _h)=M_2(G)$, then Eq. 1 reduced in the following form, which is called the second Zagreb Entropy.
  10 $$\begin{aligned} ENT_{M_2(G)}=log[M_2(G)]-\frac{1}{M_2(G)}\sum _{i=1}^{r}U_ilog[\nu _g\times \nu _h]^{[\nu _g\times \nu _h]}. \end{aligned}$$
- *First Redefined Zagreb Entropy:* If $TI=\sum _{gh\in E(G)}{\frac{\nu _g+ \nu _h}{\nu _g\times \nu _h}}=ReZG_1(G)$, then Eq. 1 is simplified in the following form and known as the first redefined Zagreb Entropy.
  11 $$\begin{aligned} ENT_{ReZG_1(G)}=log[ReZG_1(G)]-\frac{1}{ReZG_1(G)}\sum _{i=1}^{r}U_ilog\bigg [\frac{\nu _g+ \nu _h}{\nu _g\times \nu _h}\bigg ]^{\big [\frac{\nu _g+ \nu _h}{\nu _g\times \nu _h}\big ]}. \end{aligned}$$
- *Second Redefined Zagreb Entropy:* If $TI=\sum _{gh\in E(G)}{\frac{\nu _g\times \nu _h}{\nu _g+ \nu _h}}=ReZG_2(G)$, then Eq. 1 is simplified in the following form and is called the second redefined Zagreb Entropy.
  12 $$\begin{aligned} ENT_{ReZG_2(G)}=log[ReZG_2(G)]-\frac{1}{ReZG_2(G)}\sum _{i=1}^{r}U_ilog\bigg [\frac{\nu _g\times \nu _h}{\nu _g+ \nu _h}\bigg ]^{\big [\frac{\nu _g\times \nu _h}{\nu _g+ \nu _h}\big ]}. \end{aligned}$$
- *Third Redefined Zagreb Entropy:* If $TI=\sum _{gh\in E(G)}(\nu _g\times \nu _h)(\nu _g+ \nu _h)=ReZG_3(G)$, then Eq. 1 reduced in the following form and is known as the third redefined Zagreb Entropy.
  13 $$\begin{aligned} ENT_{ReZG_3(G)}=log[ReZG_3(G)]-\frac{1}{ReZG_3(G)}\sum _{i=1}^{r}U_ilog\big [(\nu _g\times \nu _h)(\nu _g+ \nu _h)\big ]^{\big [(\nu _g\times \nu _h)(\nu _g+ \nu _h)\big ]}. \end{aligned}$$
- *Atom Bond Connectivity Entropy:* If $TI=\sum _{gh\in E(G)}\sqrt{\frac{\nu _g+\nu _h-2}{\nu _g \times \nu _h}}=ABC(G)$, then Eq. 1 simplified in the following form and is known as the atom bond connectivity Entropy.
  14 $$\begin{aligned} ENT_{ABC(G)}=log[ABC(G)]-\frac{1}{ABC(G)}\sum _{i=1}^{r}U_ilog\bigg [\sqrt{\frac{\nu _g+\nu _h-2}{\nu _g \times \nu _h}}\bigg ]^{\bigg [\sqrt{\frac{\nu _g+\nu _h-2}{\nu _g \times \nu _h}}\bigg ]}. \end{aligned}$$
- *Geometric Arithmetic Entropy:* If $TI=\sum _{gh\in E(G)}{\frac{2\sqrt{\nu _g \times \nu _h}}{\nu _g+\nu _h}}=GA(G)$, then Eq. 1 reduced in the following form which is called the geometric arithmetic Entropy.
  15 $$\begin{aligned} ENT_{GA(G)}=log[GA(G)]-\frac{1}{GA(G)}\sum _{i=1}^{r}U_ilog\bigg [\frac{2\sqrt{\nu _g \times \nu _h}}{\nu _g+\nu _h}\bigg ]^{\bigg [\frac{2\sqrt{\nu _g \times \nu _h}}{\nu _g+\nu _h}\bigg ]}. \end{aligned}$$

## Polyester

The word polyester derives from the roots poly (meaning “many”) and ester (a fundamental organic compound). The main component used to produce polyester is ethylene, which is derived from petroleum. In the process, ethylene acts as the polymer, and the method that forms the finished polyester is called polymerization. Polyesters are synthetic polymers composed of many identical repeating chemical units covalently bonded by ester groups. These polymers are created by combining pure terephthalic acid and ethylene glycol, both of which are derived from petroleum. This combination produces polyethylene terephthalate, the same material used to make plastic soda bottles. The production of most polyesters involves a condensation reaction between an organic alcohol (containing hydroxyl groups) and a carboxylic acid (containing carboxyl groups). The ester linkage is a chemical group formed by these two functional groups, represented structurally by their interaction^30,31^. Polyesters have numerous properties and applications in everyday life. Common products made from polyesters include rubber tires, coloured rubber tires, compact discs, single-use soft drink bottles, and permanent-press textiles.

**Fig. 1:**
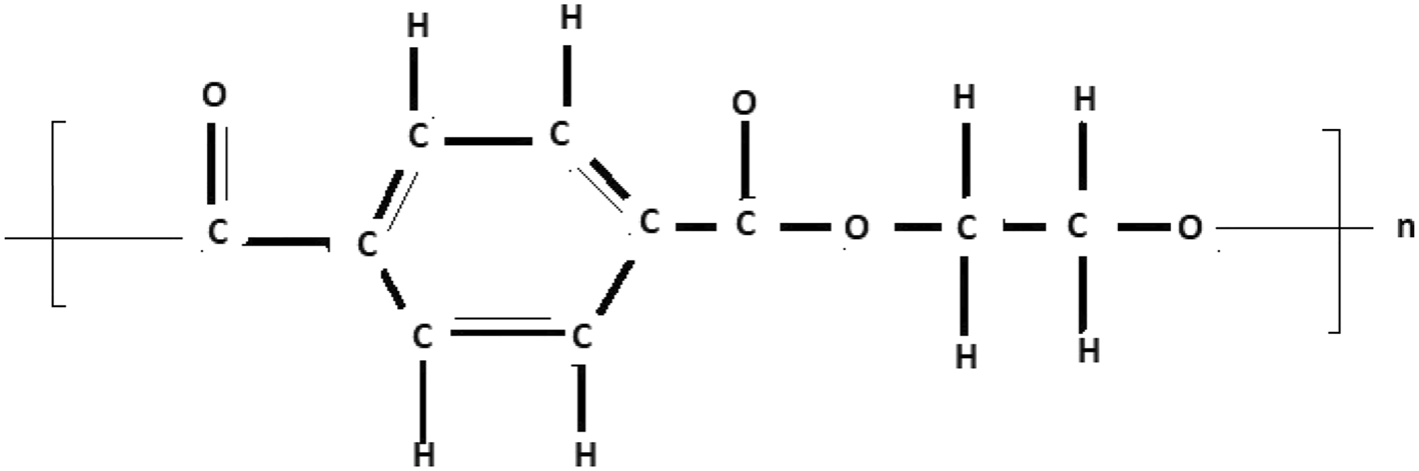
A polyester.

**Fig. 2:**
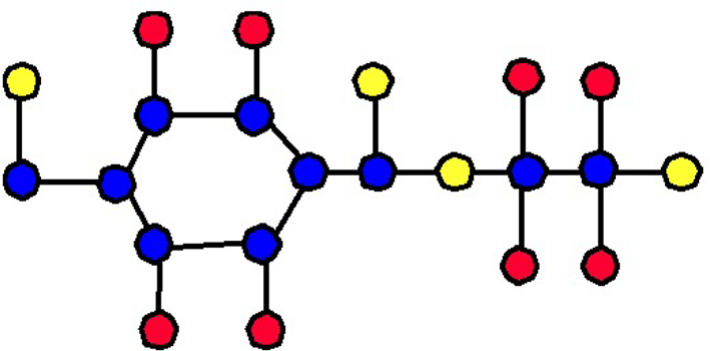
Polyester graph of one unit.

**Fig. 3:**
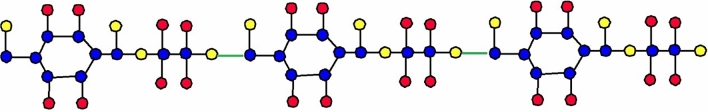
Graph with three connecting units of polyester.

**Fig. 4:**
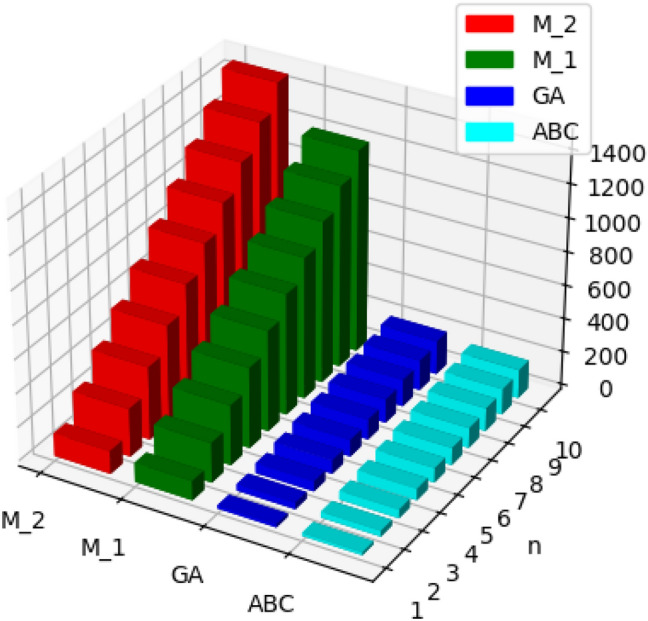
Graphical representation of $M_1, M_2, ABC, GA$ of polyester.

**Fig. 5:**
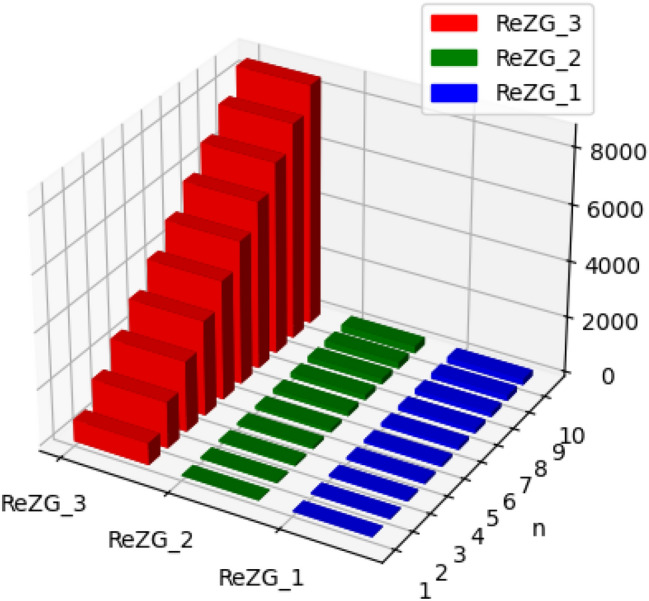
Graphical representation of $ReZG_1, ReZG_2, ReZG_3$ of polyester.

**Fig. 6:**
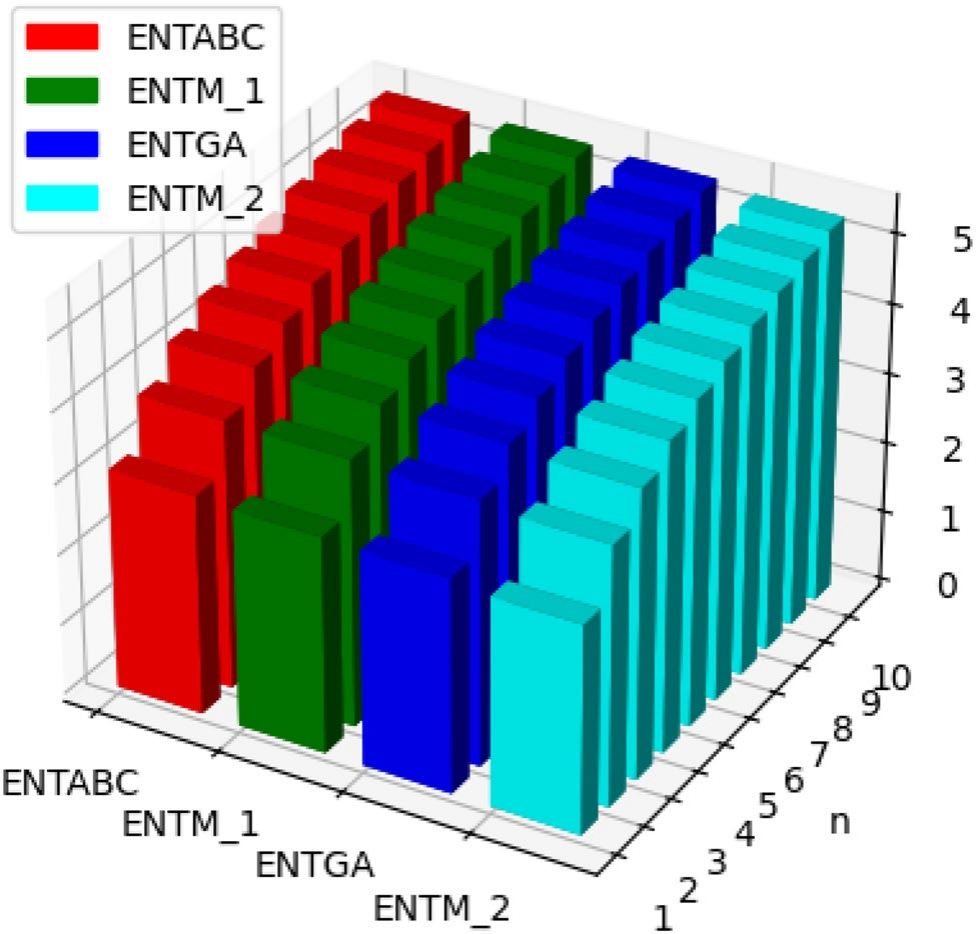
Graphical representation of $ENTM_1, ENTM_2, ENTABC, ENTGA$ of polyester.

**Fig. 7:**
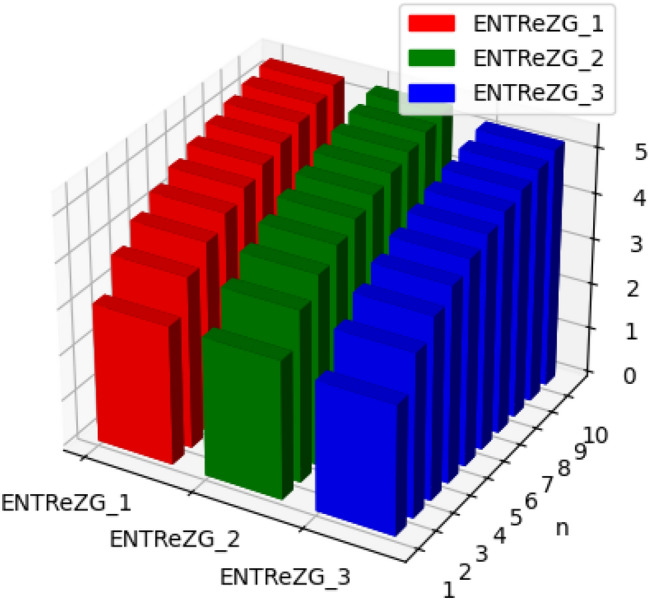
Graphical representation of $ENTReZG_1, ENTReZG_2, ENTReZG_3$ of polyester.

The molecular structure of polyester can be described in terms of atoms and bonds within its repeating unit. In a unit cell comprising n terms, the atoms are represented by vertices, and the bonds between them are represented by edges. The chemical structure of a polyester is shown in Fig. 1. The Fig. 2 shows the polyester graph for $n=1$ unit and Fig. 3 shows graph with 3 units, where blue vertices represents carbons, red vertices represents hydrogens, yellow vertices represents as oxygens and green edges used as a connection from one unit to another. Let we have a graph *G* of connection of *n* units of polyester graph then in this graphical structure there are 22*n* vertices and $23n-1$, $n\ge 1$. The edge partition for degree-based topological indices of this is presented in Table 1. The numerical values of indices and Entropies for value of *n* from 1 to 10 are shown in Table 2 and Table 3 respectively and the graphical representation of polyester for indices and Entropies are represent in Figs. 4, 5, 6 and 7.

**Table 1:** Degree-based Edge Partition of Polyester for all $n\ge 1.$.

$(\nu _g,\nu _h)$	Frequency	$(\nu _g,\nu _h)$	Frequency
(1,2)	1	(2,4)	$$2n-1$$
(1,3)	$6n-1$	(3,3)	$8n-1$
(1,4)	$4n+1$	(4,4)	*n*
(2,3)	2*n*		

### **Theorem 5.1**

*Let G represent the graphical structure depicting the connectivity of n polyester units, Then*

- $$M_1(G)=122n-8$$
- $$M_2(G)=150n-14$$
- $$ReZG_1(G)=22n$$
- $$ReZG_2(G)=26.766n-2.116$$
- $$ReZG_3(G)=868n-88$$
- $$ABC(G)=17.137n+0.0617$$
- $$GA(G)=20.287n-1.066$$

### Proof

The Zagreb indices, redefined Zagreb indices, atom bond connectivity index and geometric arithmetic index of *G* can be computed by using Table 1 and Eqs. 2, 3, 4, 5, 6, 7 and 8

- *First Zagreb Index:* $$M_{1} (G) = (1)(1 + 2) + (6n - 1)(1 + 3)$$$$+ (4n + 1)(1 + 4) + (2n)(2 + 3)$$$$+ (2n - 1)(2 + 4) + (8n - 1)(3 + 3)$$$$+ (n)(4 + 4)$$ $$M_1(G)=122n-8$$
- *Second Zagreb Index:* $$M_2(G) =(1)(1\times 2)+(6n-1)(1\times 3)$$$$+(4n+1)(1\times 4)+(2n)(2\times 3)$$$$+(2n-1)(2\times 4)+(8n-1)(3\times 3)+(n)(4\times 4)$$ $$M_2(G)=150n-14$$
- *First Redefined Zagreb Index:* $$ReZG_1(G) =(1)\frac{1+2}{1\times 2}+(6n-1)\frac{1+3}{1\times 3}$$$$+(4n+1)\frac{1+4}{1\times 4}+(2n)\frac{2+3}{2\times 3}$$$$+(2n-1)\frac{2+4}{2\times 4}+(8n-1)\frac{3+3}{3\times 3}+(n)\frac{4+4}{4\times 4}$$ $$ReZG_1(G)=22n$$
- *Second Redefined Zagreb Index:* $$ReZG_2(G) =(1)\frac{1\times 2}{1+2}+(6n-1)\frac{1\times 3}{1+3}$$$$+(4n+1)\frac{1\times 4}{1+4}+(2n)\frac{2\times 3}{2+3}$$$$+(2n-1)\frac{2\times 4}{2+4}+(8n-1)\frac{3\times 3}{3+3}+(n)\frac{4\times 4}{4+4}$$ $$ReZG_2(G)=26.766n-2.116$$
- *Third Redefined Zagreb Index:* $$ReZG_3(G) =(1)(1\times 2)(1+2)+(6n-1)(1\times 3)(1+3)$$$$+(4n+1)(1\times 4)(1+4)+(2n)(2\times 3)(2+3) +(2n-1)(2\times 4)(2+4) +(8n-1)(3\times 3)(3+3)$$$$+(n)(4\times 4)(4+4)$$ $$ReZG_3(G)=868n-88$$
- *Atom Bond Connectivity Index:* $$ABC(G) = (1)\sqrt {\frac{{1 + 2 - 2}}{{1 \times 2}}}$$$$+ (6n - 1)\sqrt {\frac{{1 + 3 - 2}}{{1 \times 3}}}$$$$+ (4n + 1)\sqrt {\frac{{1 + 4 - 2}}{{1 \times 4}}}$$$$+ (2n)\sqrt {\frac{{2 + 3 - 2}}{{2 \times 3}}}$$$$+ (2n - 1)\sqrt {\frac{{2 + 4 - 2}}{{2 \times 4}}}$$$$+ (8n - 1)\sqrt {\frac{{3 + 3 - 2}}{{3 \times 3}}}$$$$+ (n)\sqrt {\frac{{4 + 4 - 2}}{{4 \times 4}}}$$ $$ABC(G)=17.137n+0.0617$$
- *Geometric Arithmetic Index:* $$GA(G) = (1)\frac{{2\sqrt {1 \times 2} }}{{1 + 2}}$$$$+ (6n - 1)\frac{{2\sqrt {1 \times 3} }}{{1 + 3}}$$$$+ (4n + 1)\frac{{2\sqrt {1 \times 4} }}{{1 + 4}}$$$$+ (2n)\frac{{2\sqrt {2 \times 3} }}{{2 + 3}}$$$$+ (2n - 1)\frac{{2\sqrt {2 \times 4} }}{{2 + 4}}$$$$+ (8n - 1)\frac{{2\sqrt {3 \times 3} }}{{3 + 3}} + (n)\frac{{2\sqrt {4 \times 4} }}{{4 + 4}}$$ $$GA(G)=20.287n-1.066$$

$$\square$$

**Table 2:** Numerical comparison of degree-based indices of polyester for $n=$ 1–10.

$n$	$M_1(G)$	$M_2(G)$	$ReZG_1(G)$	$ReZG_2(G)$	$ReZG_3(G)$	$ABC(G)$	$GA(G)$
1	114	136	22	24.65	780	17.1987	19.221
2	236	286	44	51.416	1648	34.3357	39.508
3	358	436	66	78.182	2516	51.4727	59.795
4	480	586	88	104.948	3384	68.6097	80.082
5	602	736	110	131.714	4252	85.7467	100.369
6	724	886	132	158.48	5120	102.8837	120.656
7	846	1036	154	185.246	5988	120.0207	140.943
8	968	1186	176	212.012	6856	137.1577	161.23
9	1090	1336	198	238.778	7724	154.2947	181.517
10	1212	1486	220	265.544	8592	171.4317	201.804

### **Theorem 5.2**

*Let G represent the graphical structure depicting the connectivity of n polyester units. Then the Entropy of indices are given by*

- $$ENT_{M_1(G)}=\frac{2(61n - 4) \log (122n - 8) - 205.6953n + 15.7033}{2(61n - 4)}$$.
- $$ENT_{M_2(G)}=\frac{2(75n - 7) \log (150n - 14) - 299.2895n + 32.7749}{2(75n - 7)}$$.
- $$ENT_{ReZG_1(G)}=\log (22n) - 0.00785 - \frac{0.04498}{n}$$.
- $$ENT_{ReZG_2(G)}=\frac{(26.766n - 2.116) \log (26.766n - 2.116) - 5.448n + 1.2248}{26.766n - 2.116}$$.
- $$ENT_{ReZG_3(G)}=\frac{4(217n - 22) \log (868n - 88) - 3338.58n + 360.38}{4(217n - 22)}$$.
- $$ENT_{ABC(G)}=\frac{(17.137n + 0.0617) \log (17.137n + 0.0617) + 4.9345n - 0.3113}{17.137n + 0.0617}$$.
- $$ENT_{GA(G)}=\frac{(20.287n - 1.066) \log (20.287n - 1.066) + 1.6125n + 0.0539}{20.287n - 1.066}$$.

### Proof

The Entropy of Zagreb, redefined Zagreb, atom bond connectivity and geometric topological indices of *G* can be computed by using Table 1 and Eqs. 9, 10, 11, 12, 13, 14 and 15

- *First Zagreb Entropy:* $$ENT_{{M_{1} (G)}} = log[122n - 8]$$$$- \frac{1}{{122n - 8}}[(1)log(1 + 2)^{{1 + 2}}$$$$+ (6n - 1)log(1 + 3)^{{(1 + 3)}}$$$$+ (4n + 1)log(1 + 4)^{{(1 + 4)}} + (2n)log(2 + 3)^{{(2 + 3)}}$$$$+ (2n - 1)log(2 + 4)^{{(2 + 4)}}$$$$+ (8n - 1)log(3 + 3)^{{(3 + 3)}}$$$$+ (n)log(4 + 4)^{{(4 + 4)}} ],$$ $$ENT_{M_1(G)}=\frac{2(61n - 4) \log (122n - 8) - 205.6953n + 15.7033}{2(61n - 4)}$$.
- *Second Zagreb Entropy:* $$ENT_{{M_{2} (G)}} = log[150n - 14] - \frac{1}{{150n - 14}}[(1)log(1 \times 2)^{{(1 \times 2)}}$$$$+ (6n - 1)log(1 \times 3)^{{(1 \times 3)}}$$$$+ (4n + 1)log(1 \times 4)^{{(1 \times 4)}}$$$$+ (2n)log(2 \times 3)^{{(2 \times 3)}}$$$$+ (2n - 1)log(2 \times 4)^{{(2 \times 4)}}$$$$+ (8n - 1)log(3 \times 3)^{{(3 \times 3)}}$$$$+ (n)log(4 \times 4)^{{(4 \times 4)}} ],$$ $$ENT_{M_2(G)}=\frac{2(75n - 7) \log (150n - 14) - 299.2895n + 32.7749}{2(75n - 7)}$$.
- *First Redefined Zagreb Entropy:* $$ENT_{{ReZG_{1} (G)}} = log[22n] - \frac{1}{{22n}}[(1)log(\frac{{1 + 2}}{{1 \times 2}})^{{(\frac{{1 + 2}}{{1 \times 2}})}}$$$$+ (6n - 1)log(\frac{{1 + 3}}{{1 \times 3}})^{{(\frac{{1 + 3}}{{1 \times 3}})}}$$$$+ (4n + 1)log[\frac{{1 + 4}}{{1 \times 4}}]^{{(\frac{{1 + 4}}{{1 \times 4}})}}$$$$+ (2n)log(\frac{{2 + 3}}{{2 \times 3}})^{{(\frac{{2 + 3}}{{2 \times 3}})}} + (2n - 1)log(\frac{{2 + 4}}{{2 \times 4}})^{{(\frac{{2 + 4}}{{2 \times 4}})}}$$$$+ (8n - 1)log(\frac{{3 + 3}}{{3 \times 3}})^{{(\frac{{3 + 3}}{{3 \times 3}})}}$$$$+ (n)log(\frac{{4 + 4}}{{4 \times 4}})^{{(\frac{{4 + 4}}{{4 \times 4}})}} ],$$ $$ENT_{ReZG_1(G)}=\log (22n) - 0.00785 - \frac{0.04498}{n}$$.
- *Second Redefined Zagreb Entropy:* $$ENT_{{ReZG_{2} (G)}} = log[26.766n - 2.116]$$$$- \frac{1}{{26.766n - 2.116}}[(1)log(\frac{{1 \times 2}}{{1 + 2}})^{{(\frac{{1 \times 2}}{{1 + 2}})}}$$$$+ (6n - 1)log(\frac{{1 \times 3}}{{1 + 3}})^{{(\frac{{1 \times 3}}{{1 + 3}})}}$$$$+ (4n + 1)log[\frac{{1 \times 4}}{{1 + 4}}]^{{(\frac{{1 \times 4}}{{1 + 4}})}}$$$$+ (2n)log(\frac{{2 \times 3}}{{2 + 3}})^{{(\frac{{2 \times 3}}{{2 + 3}})}}$$$$+ (2n - 1)log(\frac{{2 \times 4}}{{2 + 4}})^{{(\frac{{2 \times 4}}{{2 + 4}})}}$$$$+ (8n - 1)log(\frac{{3 \times 3}}{{3 + 3}})^{{(\frac{{3 \times 3}}{{3 + 3}})}}$$$$+ (n)log(\frac{{4 \times 4}}{{4 + 4}})^{{(\frac{{4 \times 4}}{{4 + 4}})}} ],$$ $$ENT_{ReZG_2(G)}=\frac{(26.766n - 2.116) \log (26.766n - 2.116) - 5.448n + 1.2248}{26.766n - 2.116}$$.
- *Third Redefined Zagreb Entropy:* $$ENT_{{ReZG_{3} (G)}} = log[868n - 88]$$$$- \frac{1}{{868n - 88}}[(1)log((1 \times 2)(1 + 2))^{{((1 \times 2)(1 + 2))}} + (6n - 1)$$$$log((1 \times 3)(1 + 3))^{{((1 \times 3)(1 + 3))}} + (4n + 1)$$$$log((1 \times 4)(1 + 4))^{{((1 \times 4)(1 + 4))}} + (2n)$$$$log((2 \times 3)(2 + 3))^{{((2 \times 3)(2 + 3))}} + (2n - 1)$$$$log((2 \times 4)(2 + 4))^{{((2 \times 4)(2 + 4))}} + (8n - 1)$$$$log((3 \times 3)(3 + 3))^{{((3 \times 3)(3 + 3))}}$$$$+ (n)log((4 \times 4)(4 + 4))^{{((4 \times 4)(4 + 4))}} ],$$ $$ENT_{ReZG_3(G)}=\frac{4(217n - 22) \log (868n - 88) - 3338.58n + 360.38}{4(217n - 22)}$$.
- *Atom Bond Connectivity Entropy:* $$ENT_{{ABC(G)}} = log[17.137n + 0.0617]$$$$- \frac{1}{{17.137n + 0.0617}}$$$$[(1)log\left( {\sqrt {\frac{{1 + 2 - 2}}{{1 \times 2}}} } \right)^{{\left( {\sqrt {\frac{{1 + 2 - 2}}{{1 \times 2}}} } \right)}}$$$$+ (6n - 1)$$$$log\left( {\sqrt {\frac{{1 + 3 - 2}}{{1 \times 3}}} } \right)^{{\left( {\sqrt {\frac{{1 + 3 - 2}}{{1 \times 3}}} } \right)}}$$$$+ (4n + 1)$$$$log\left( {\sqrt {\frac{{1 + 4 - 2}}{{1 \times 4}}} } \right)^{{\left( {\sqrt {\frac{{1 + 4 - 2}}{{1 \times 4}}} } \right)}} + (2n)$$$$log(\sqrt {\frac{{2 + 3 - 2}}{{2 \times 3}}} (^{{(\sqrt {\frac{{2 + 3 - 2}}{{2 \times 3}}} )}} + (2n - 1)$$$$log(\sqrt {\frac{{2 + 4 - 2}}{{2 \times 4}}} )^{{(\sqrt {\frac{{2 + 4 - 2}}{{2 \times 4}}} )}} + (8n - 1)$$$$log\left( {\sqrt {\frac{{3 + 3 - 2}}{{3 \times 3}}} } \right)^{{\left( {\sqrt {\frac{{3 + 3 - 2}}{{3 \times 3}}} } \right)}}$$$$+ (n)log\left( {\sqrt {\frac{{4 + 4 - 2}}{{4 \times 4}}} } \right)^{{\left( {\sqrt {\frac{{4 + 4 - 2}}{{4 \times 4}}} } \right)}} ],$$ $$ENT_{ABC(G)}=\frac{(17.137n + 0.0617) \log (17.137n + 0.0617) + 4.9345n - 0.3113}{17.137n + 0.0617}$$.
- *Geometric Arithmetic Entropy:* $$ENT_{{GA(G)}} = log[20.287n - 1.066]$$$$- \frac{1}{{20.287n - 1.066}}$$$$[(1)log\left( {\frac{{2\sqrt {1 \times 2} }}{{1 + 2}}} \right)^{{\left( {\frac{{2\sqrt {1 \times 2} }}{{1 + 2}}} \right)}}$$$$+ (6n - 1)log\left( {\frac{{2\sqrt {1 \times 3} }}{{1 + 3}}} \right)^{{\left( {\frac{{2\sqrt {1 \times 3} }}{{1 + 3}}} \right)}}$$$$+ (4n + 1)log\left( {\frac{{2\sqrt {1 \times 4} }}{{1 + 4}}} \right)^{{\left( {\frac{{2\sqrt {1 \times 4} }}{{1 + 4}}} \right)}}$$$$+ (2n)log\left( {\frac{{2\sqrt {2 \times 3} }}{{2 + 3}}} \right)^{{\left( {\frac{{2\sqrt {2 \times 3} }}{{2 + 3}}} \right)}}$$$$+ (2n - 1)log\left( {\frac{{2\sqrt {2 \times 4} }}{{2 + 4}}} \right)^{{\left( {\frac{{2\sqrt {2 \times 4} }}{{2 + 4}}} \right)}}$$$$+ (8n - 1)log[\left( {\frac{{2\sqrt {3 \times 3} }}{{3 + 3}}} \right)^{{\left( {\frac{{2\sqrt {3 \times 3} }}{{3 + 3}}} \right)}}$$$$+ (n)log\left( {\frac{{2\sqrt {4 \times 4} }}{{4 + 4}}} \right)^{{\left( {\frac{{2\sqrt {4 \times 4} }}{{4 + 4}}} \right)}} ],$$ $$ENT_{GA(G)}=\frac{(20.287n - 1.066) \log (20.287n - 1.066) + 1.6125n + 0.0539}{20.287n - 1.066}$$.

$$\square$$

**Table 3:** Numerical Comparison of Degree-based Entropies of Polyester for *n*= 1 to 10.

$n$	$ENT_{M_1}$	$ENT_{M_2}$	$ENT_{ReZG_1}$	$ENT_{ReZG_2}$	$ENT_{ReZG_3}$	$ENT_{ABC}$	$ENT_{GA}$
1	3.07	2.952	3.038	3.033	2.841	3.114	3.042
2	3.787	3.677	3.753	3.751	3.574	3.815	3.759
3	4.201	4.093	4.166	4.165	3.992	4.223	4.172
4	4.492	4.386	4.458	4.457	4.287	4.512	4.4643
5	4.718	4.612	4.683	4.682	4.514	4.736	4.689
6	4.902	4.796	4.867	4.866	4.698	4.918	4.873
7	5.057	4.952	5.022	5.021	4.854	5.073	5.028
8	5.191	5.087	5.157	5.156	4.989	5.207	5.163
9	5.310	5.206	5.275	5.275	5.108	5.325	5.281
10	5.416	5.311	5.381	5.380	5.214	5.430	5.387

## Polycarbonate

Polycarbonate is a strong, transparent thermoplastic known for its high impact resistance and optical clarity, making it widely used in various industries. It is primarily produced from bisphenol (BPA) and phosgene through interfacial polycondensation or transesterification processes. The polymer’s amorphous structure and repeating units contribute to its transparency and strength. Polycarbonate finds applications in electronics, eyewear, automotive parts, construction materials, medical devices, and packaging due to its durability and clarity. Its high impact resistance and thermal stability make it suitable for safety and security applications, such as bullet-resistant windows and medical equipment housings. However, concerns about the potential health risks of BPA and its susceptibility to scratching are notable drawbacks. Advances in coatings and composites aim to enhance polycarbonate’s properties, while sustainability efforts are focused on developing BPA-free alternatives and improving recycling methods. Despite these challenges, polycarbonate remains an important material in modern manufacturing, balancing performance with ongoing innovations to address environmental and health concerns^32,33^.

**Fig. 8:**
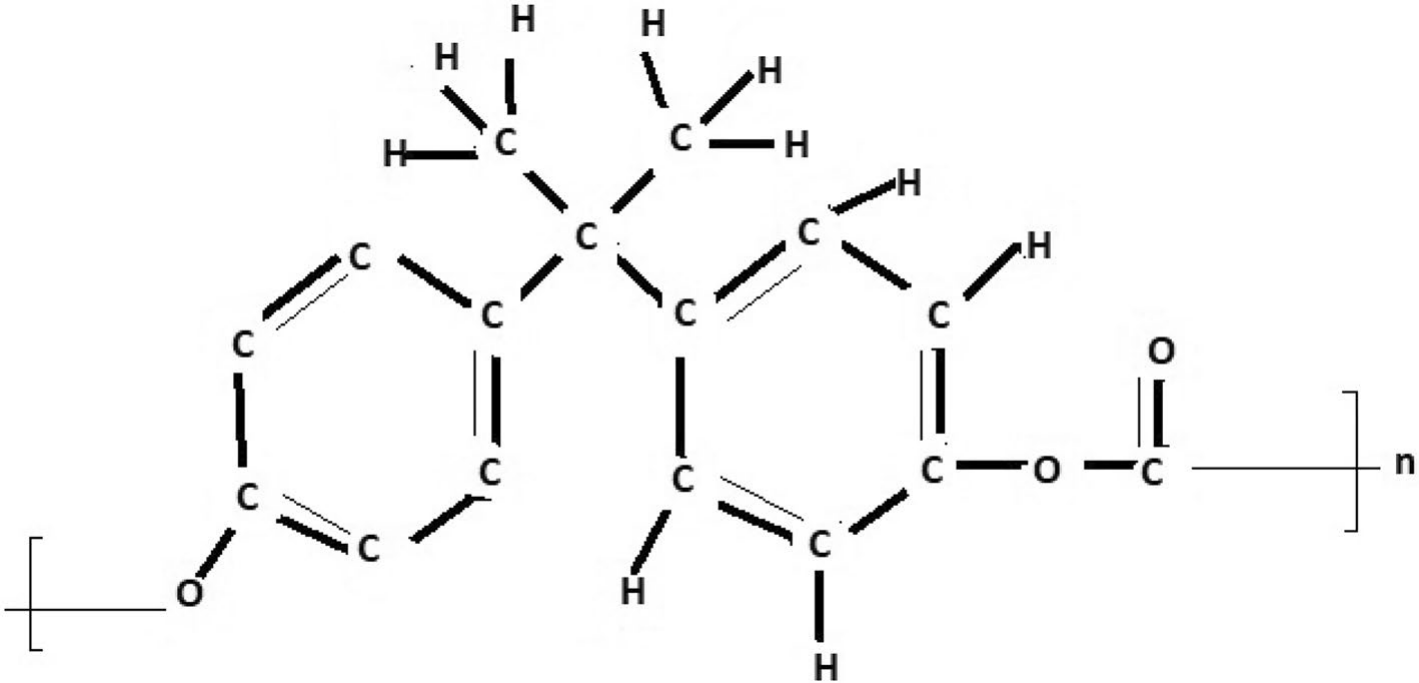
A polycarbonate.

**Fig. 9:**
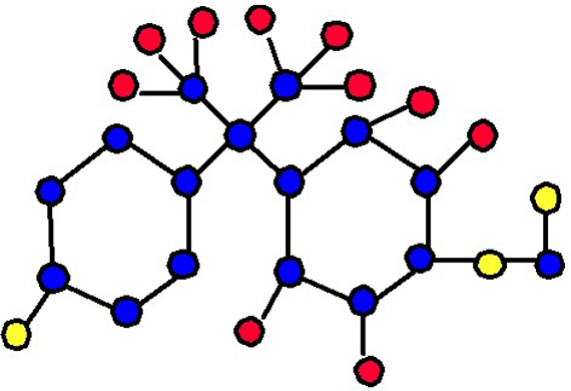
Polycarbonate graph of one unit.

**Fig. 10:**
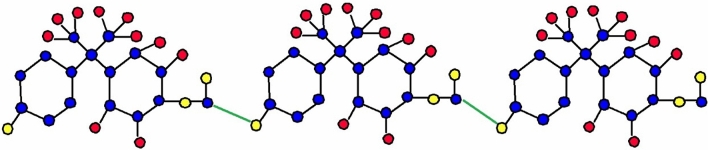
Graph with three connecting units of polycarbonate.

**Fig. 11:**
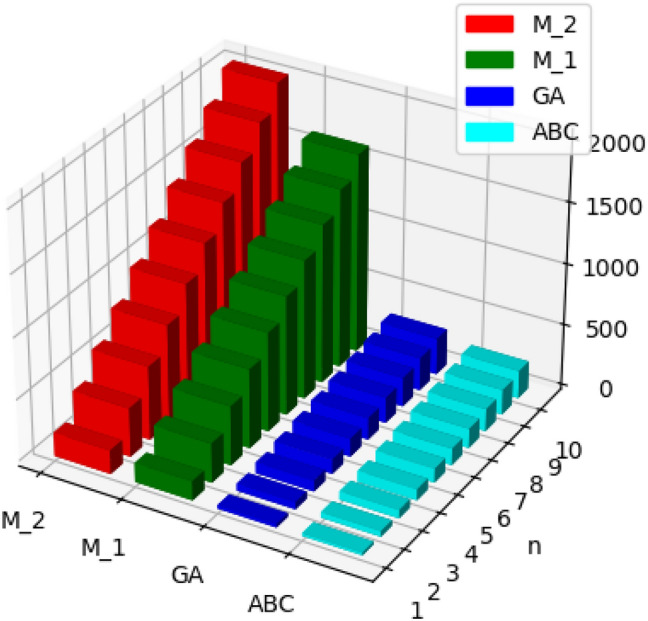
Graphical representation of $M_1, M_2, ABC, GA$ of polycarbonate.

**Fig. 12:**
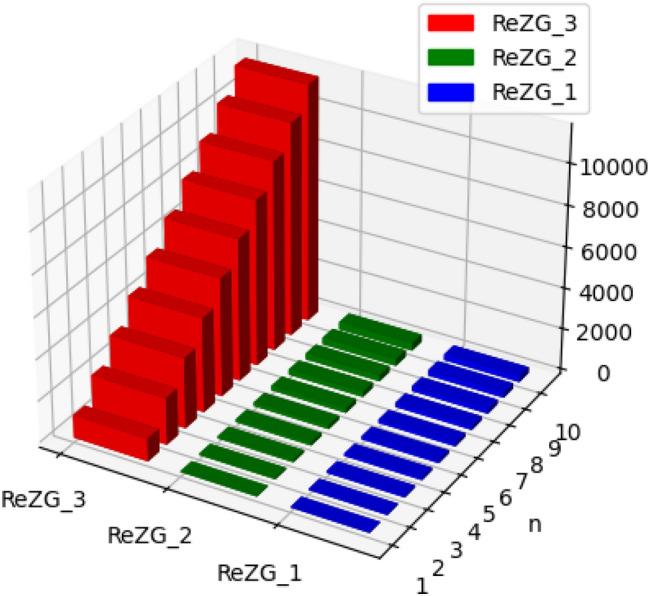
Graphical representation of $ReZG_1, ReZG_2, ReZG_3$ of polycarbonate.

**Fig. 13:**
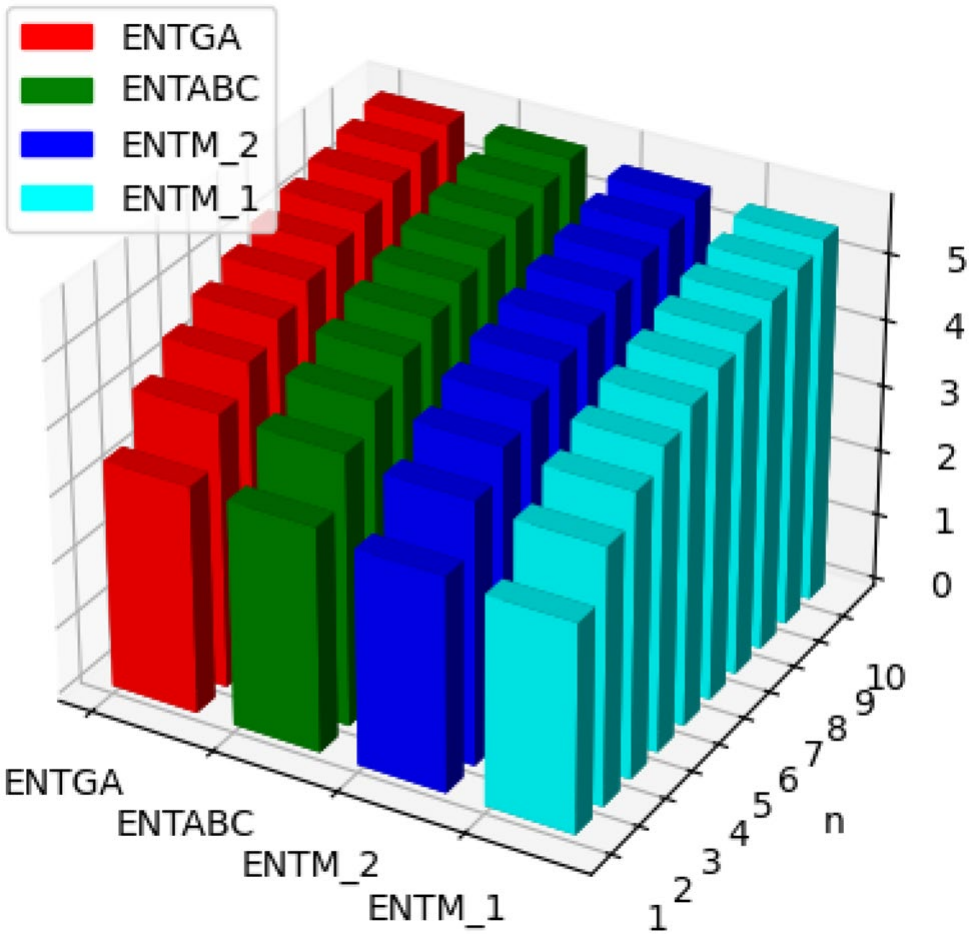
Graphical representation of $ENTM_1, ENTM_2, ENTABC, ENTGA$ of polycarbonate.

**Fig. 14:**
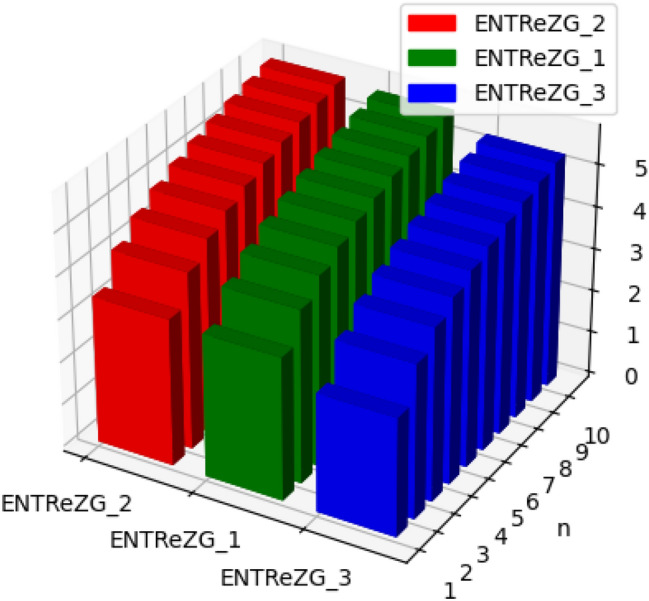
Graphical representation of $ENTReZG_1, ENTReZG_2, ENTReZG_3$ of polycarbonate.

The chemical structure of polycarbonate with one unit cell containing atoms and bonds is illustrated in Fig. 8. The Fig. 9 shows the polycarbonate graph for $n=1$ unit and Fig. 10 shows graph with 3 units, where blue vertices represents carbons, red vertices represents hydrogens, yellow vertices represents as oxygens and green edges used as a connection from one unit to another. Let we have a graph *G* of connection of *n* units of polycarbonate graph then in this graphical structure there are 29*n* vertices and $31n-1$, $n\ge 1$. The edge partitioning of this graph *G* for degree-based topological indices is shown in Table 4. The numerical values of indices and Entropies for value of *n* from 1 to 10 are shows in Tables 5 and 6 respectively and the graphical representation of indices and Entropies of this graph are represent in Figs. 11, 12, 13 and 14.

**Table 4:** Degree-based edge partition of polycarborate for all $n\ge 1.$.

$(\nu _g,\nu _h)$	Frequency	$(\nu _g,\nu _h)$	Frequency
(1,2)	1	(2,3)	$$8n-3$$
(1,3)	5*n*	(3,3)	6*n*
(1,4)	6*n*	(3,4)	2*n*
(2,2)	$2n+1$	(4,4)	2*n*

### **Theorem 6.1**

Let G represent the graphical structure depicting the connectivity of n units of polycarbonate, then

- $$M_1(G)=164n-8$$.
- $$M_2(G)=205n-12$$.
- $$ReZG_1(G)=29n$$.
- $$ReZG_2(G)=36.578n-1.93$$.
- $$ReZG_3(G)=1168n-68$$.
- $$ABC(G)=22.865n-0.707$$.
- $$GA(G)=30.027n-0.996$$.

### Proof

The Zagreb, redefined Zagreb, atom bond connectivity and geometric topological indices of graph *G* can be computed by using Table 4 and Eqs. 2, 3, 4, 5, 6, 7 and 8

- *First Zagreb Index:* $$M_1(G) =(1)(1+2)+(5n)(1+3)$$$$+(6n)(1+4)+(2n+1)(2+2)$$$$+(8n-3)(2+3)+(6n)(3+3)+(2n)(3+4)+(2n)(4+4)$$ $$M_1(G)=164n-8$$.
- *Second Zagreb Index:* $$M_2(G) =(1)(1\times 2)+(5n)(1\times 3)+(6n)(1\times 4)$$$$+(2n+1)(2\times 2) +(8n-3)(2\times 3)$$$$+(6n)(3\times 3)+(2n)(3\times 4)+(2n)(4\times 4)$$ $$M_2(G)=205n-12$$.
- *First Redefined Zagreb Index:* $$ReZG_1(G) =(1)\frac{1+2}{1\times 2}+(5n)\frac{1+3}{1\times 3}$$$$+(6n)\frac{1+4}{1\times 4}+(2n+1)\frac{2+2}{2\times 2}+(8n-3)\frac{2+3}{2\times 3}$$$$+(6n)\frac{3+3}{3\times 3}+(2n)\frac{3+4}{3\times 4}+(2n)\frac{4+4}{4\times 4}$$ $$ReZG_1(G)=29n$$.
- *Second Redefined Zagreb Index:* $$ReZG_2(G) =(1)\frac{1\times 2}{1+2}+(5n)\frac{1\times 3}{1+3}$$$$+(6n)\frac{1\times 4}{1+4}+(2n+1)\frac{2\times 2}{2+2}$$$$+(8n-3)\frac{2\times 3}{2+3} +(6n)\frac{3\times 3}{3+3}$$$$+(2n)\frac{3\times 4}{3+4}+(2n)\frac{4\times 4}{4+4}$$ $$ReZG_2(G)=36.578n-1.93$$.
- *Third Redefined Zagreb Index:* $$ReZG_3(G) =(1)(1\times 2)(1+2)+(5n)(1\times 3)(1+3)$$$$+(6n)(1\times 4)(1+4)+(2n+1)(2\times 2)(2+2)+(8n-3)(2\times 3)(2+3)$$$$+(6n)(3\times 3)(3+3)+(2n)(3\times 4)(3+4)+(2n)(4\times 4)(4+4)$$ $$ReZG_3(G)=1168n-68$$
- *Atom Bond Connectivity Index:* $$ABC(G) =(1)\sqrt{\frac{1+2-2}{1 \times 2}}+(5n)\sqrt{\frac{1+3-2}{1 \times 3}}$$$$+(6n)\sqrt{\frac{1+4-2}{1 \times 4}}$$$$+(2n+1)\sqrt{\frac{2+2-2}{2 \times 2}}$$$$+(8n-3)\sqrt{\frac{2+3-2}{2 \times 3}}$$$$+(6n)\sqrt{\frac{3+3-2}{3 \times 3}}$$$$+(2n)\sqrt{\frac{3+4-2}{3 \times 4}}+(2n)\sqrt{\frac{4+4-2}{4 \times 4}}$$ $$ABC(G)=22.865n-0.707$$.
- *Geometric Arithmetic Index:* $$GA(G) =(1)\frac{2\sqrt{1\times 2}}{1+2}+(5n)\frac{2\sqrt{1\times 3}}{1+3}$$$$+(6n)\frac{2\sqrt{1\times 4}}{1+4}+(2n+1)\frac{2\sqrt{2\times 2}}{2+2}$$$$+(8n-3)\frac{2\sqrt{2\times 3}}{2+3}+(6n)\frac{2\sqrt{3\times 3}}{3+3}$$$$+(2n)\frac{2\sqrt{3\times 4}}{3+4}+(2n)\frac{2\sqrt{4\times 4}}{4+4}$$ $$GA(G)=30.027n-0.996$$.

$$\square$$

**Table 5:** Numerical comparison of degree-based indices of polycarbonate for *n* = 1–10.

*n*	$M_1(G)$	$M_2(G)$	$ReZG_1(G)$	$ReZG_2(G)$	$ReZG_3(G)$	ABC(G)	GA(G)
1	156	193	29	34.65	1100	22.158	29.031
2	320	398	58	71.226	2268	45.023	59.058
3	484	603	87	107.804	3436	67.888	89.085
4	648	808	116	144.382	4604	90.753	119.112
5	812	1013	145	180.96	5772	113.618	149.139
6	976	1218	174	217.538	6940	136.483	179.166
7	1140	1423	203	254.116	8108	159.348	209.193
8	1304	1628	232	290.694	9276	182.213	239.22
9	1468	1833	261	327.272	10444	205.078	269.247
10	1632	2038	290	363.85	11612	227.943	299.274

### **Theorem 6.2**

*Let G represent the graphical structure depicting the connectivity of n units of polycarbonate. Then, the Entropy of the topological indices are given by*

- $$ENT_{M_1(G)}=\frac{4(41n - 2) \log (164n - 8) - 311.1514n + 15.3006}{4(41n - 2)}$$.
- $$ENT_{M_2(G)}=\frac{(205n - 12) \log (205n - 12) - 413.856n + 25.3202}{205n - 12}$$.
- $$ENT_{ReZG_1(G)}=\log (29n) + 0.01958 - \frac{0.03669}{n}$$.
- $$ENT_{ReZG_2(G)}=\frac{(36.578n - 1.93) \log (36.578n - 1.93) - 7.8702n + 0.9267}{36.578n - 1.93}$$.
- $$ENT_{ReZG_3(G)}=\frac{4(292n - 17) \log (1168n - 68) - 4692.52n + 46.924}{4(292n - 17)}$$.
- $$ENT_{ABC(G)}=\frac{(22.865n - 0.707) \log (22.865n - 0.707) + 6.8133n - 0.2451}{22.865n - 0.707}$$.
- $$ENT_{GA(G)}=ENT(G) = \frac{(30.027n - 0.996) \log (30.027n - 0.996) + 1.8743n - 0.0045}{30.027n - 0.996}$$.

### Proof

The Entropy of Zagreb indices, redefined Zagreb indices, atom bond connectivity index and geometric arithmetic index of *n* connecting units of polycarbonate structure can be computed by using Table 4 and Eqs. 9, 10, 11, 12, 13, 14 and 15.

- *First Zagreb Entropy:* $$ENT_{{M_{1} (G)}} = log[164n - 8]$$$$- \frac{1}{{164n - 8}}[(1)log(1 + 2)^{{(1 + 2)}}$$$$+ (5n)log((1 + 3)^{{(1 + 3)}}$$$$+ (6n)log(1 + 4)^{{(1 + 4)}}$$$$+ (2n + 1)log(2 + 2)^{{(2 + 2)}}$$$$+ (8n - 3)log(2 + 3)^{{(2 + 3)}}$$$$+ (6n)log(3 + 3)^{{(3 + 3)}}$$ $$ENT_{M_1(G)}=\frac{4(41n - 2) \log (164n - 8) - 311.1514n + 15.3006}{4(41n - 2)}$$.
- *Second Zagreb Entropy:* $$ENT_{{M_{2} (G)}} = log[205n - 12]$$$$- \frac{1}{{205n - 12}}[(1)log(1 \times 2)^{{(1 \times 2)}} + (5n)$$$$log(1 \times 3)^{{(1 \times 3)}} + (6n)$$$$log(1 \times 4)^{{(1 \times 4)}} + (2n + 1)$$$$log(2 \times 2)^{{(2 \times 2)}} + (8n - 3)$$$$log(2 \times 3)^{{(2 \times 3)}} + (6n)$$$$log(3 \times 3)^{{(3 \times 3)}} + (2n)log(3 \times 4)^{{(3 \times 4)}} + (2n)$$$$log(4 \times 4)^{{(4 \times 4}} ],$$ $$ENT_{M_2(G)}=\frac{(205n - 12) \log (205n - 12) - 413.856n + 25.3202}{205n - 12}$$.
- *First Redefined Zagreb Entropy:* $$ENT_{{ReZG_{1} (G)}} = log[29n]$$$$- \frac{1}{{29n}}[(1)log(\frac{{1 + 2}}{{1 \times 2}})^{{(\frac{{1 + 2}}{{1 \times 2}})}}$$$$+ (5n)log(\frac{{1 + 3}}{{1 \times 3}})^{{(\frac{{1 + 3}}{{1 \times 3}})}}$$$$+ (6n)log(\frac{{1 + 4}}{{1 \times 4}})^{{(\frac{{1 + 4}}{{1 \times 4}})}}$$$$+ (2n + 1)log(\frac{{2 + 2}}{{2 \times 2}})^{{(\frac{{2 + 2}}{{2 \times 2}})}}$$$$+ (8n - 3)log(\frac{{2 + 3}}{{2 \times 3}})^{{(\frac{{2 + 3}}{{2 \times 3}})}}$$$$+ (6n)log(\frac{{3 + 3}}{{3 \times 3}})^{{(\frac{{3 + 3}}{{3 \times 3}})}}$$$$+ (2n)log(\frac{{3 + 4}}{{3 \times 4}})^{{(\frac{{3 + 4}}{{3 \times 4}})}}$$$$+ (2n)log(\frac{{4 + 4}}{{4 \times 4}})^{{(\frac{{4 + 4}}{{4 \times 4}})}} ],$$ $$ENT_{ReZG_1(G)}=\log (29n) + 0.01958 - \frac{0.03669}{n}$$.
- *Second Redefined Zagreb Entropy:* $$ENT_{{ReZG_{2} (G)}} = log[36.578n - 1.93]$$$$- \frac{1}{{36.578n - 1.93}}[(1)log(\frac{{1 \times 2}}{{1 + 2}})^{{(\frac{{1 \times 2}}{{1 + 2}})}}$$$$+ (5n)log(\frac{{1 \times 3}}{{1 + 3}})^{{(\frac{{1 \times 3}}{{1 + 3}})}}$$$$+ (6n)log(\frac{{1 \times 4}}{{1 + 4}})^{{(\frac{{1 \times 4}}{{1 + 4}})}}$$$$+ (2n + 1)log(\frac{{2 \times 2}}{{2 + 2}})^{{(\frac{{2 \times 2}}{{2 + 2}})}}$$$$+ (8n - 3)log(\frac{{2 \times 3}}{{2 + 3}})^{{(\frac{{2 \times 3}}{{2 + 3}})}}$$$$+ (6n)log(\frac{{3 \times 3}}{{3 + 3}})^{{(\frac{{3 \times 3}}{{3 + 3}})}} + (2n)log(\frac{{3 \times 4}}{{3 + 4}})^{{(\frac{{3 \times 4}}{{3 + 4}})}}$$$$+ (2n)log(\frac{{4 \times 4}}{{4 + 4}})^{{(\frac{{4 \times 4}}{{4 + 4}})}} ],$$ $$ENT_{ReZG_2(G)}=\frac{(36.578n - 1.93) \log (36.578n - 1.93) - 7.8702n + 0.9267}{36.578n - 1.93}$$.
- *Third Redefined Zagreb Entropy:* $$ENT_{{ReZG_{3} (G)}} = log[1168n - 68]$$$$- \frac{1}{{1168n - 68}}[(1)log((1 \times 2)(1 + 2))^{{((1 \times 2)(1 + 2))}} + (5n)log((1 \times 3)(1 + 3))^{{((1 \times 3)(1 + 3))}}$$$$+ (6n)log((1 \times 4)(1 + 4))^{(} (1 \times 4)(1 + 4)) + (2n + 1)log((2 \times 2)(2 + 2))^{{((2 \times 2)(2 + 2))}}$$$$+ (8n - 1)log((2 \times 3)(2 + 3))^{{((2 \times 3)(2 + 3))}}$$$$+ (6n)log((3 \times 3)(3 + 3))^{{((3 \times 3)(3 + 3))}}$$$$+ (2n)log((3 \times 4)(3 + 4))^{{((3 \times 4)(3 + 4))}}$$$$+ (2n)log((4 \times 4)(4 + 4))^{{((4 \times 4)(4 + 4))}} ],$$ $$ENT_{ReZG_3(G)}=\frac{4(292n - 17) \log (1168n - 68) - 4692.52n + 46.924}{4(292n - 17)}$$.
- *Atom Bond Connectivity Entropy:* $$ENT_{{ABC(G)}} = log[22.865n - 0.707]$$$$- \frac{1}{{22.865n - 0.707}}$$$$[(1)log\left( {\sqrt {\frac{{1 + 2 - 2}}{{1 \times 2}}} } \right)^{{\left( {\sqrt {\frac{{1 + 2 - 2}}{{1 \times 2}}} } \right)}}$$$$+ (5n)log$$$$\left( {\sqrt {\frac{{1 + 3 - 2}}{{1 \times 3}}} } \right)^{{\left( {\sqrt {\frac{{1 + 3 - 2}}{{1 \times 3}}} } \right)}} + (6n)log \left( {\sqrt {\frac{{1 + 4 - 2}}{{1 \times 4}}} } \right)^{{\left( {\sqrt {\frac{{1 + 4 - 2}}{{1 \times 4}}} } \right)}}$$$$+ (2n + 1)log$$$$\left( {\sqrt {\frac{{2 + 2 - 2}}{{2 \times 2}}} } \right)^{{\left( {\sqrt {\frac{{2 + 2 - 2}}{{2 \times 2}}} } \right)}}$$$$+ (8n - 3)log$$$$\left( {\sqrt {\frac{{2 + 3 - 2}}{{2 \times 3}}} } \right)^{{\left( {\sqrt {\frac{{2 + 3 - 2}}{{2 \times 3}}} } \right)}}$$$$+ (6n)log$$$$\left( {\sqrt {\frac{{3 + 3 - 2}}{{3 \times 3}}} } \right)^{{\left( {\sqrt {\frac{{3 + 3 - 2}}{{3 \times 3}}} } \right)}}$$$$+ (2n)log$$$$\left( {\sqrt {\frac{{3 + 4 - 2}}{{3 \times 4}}} } \right)^{{\left( {\sqrt {\frac{{3 + 4 - 2}}{{3 \times 4}}} } \right)}}$$$$+ (2n)log$$$$\left( {\sqrt {\frac{{4 + 4 - 2}}{{4 \times 4}}} } \right)^{{\left( {\sqrt {\frac{{4 + 4 - 2}}{{4 \times 4}}} } \right)}} ],$$ $$ENT_{ABC(G)}=\frac{(22.865n - 0.707) \log (22.865n - 0.707) + 6.8133n - 0.2451}{22.865n - 0.707}$$.
- *Geometric Arithmetic Entropy:* $$ENT_{{GA(G)}} = log[30.027n - 0.996]$$$$- \frac{1}{{30.027n - 0.996}}$$$$[(1)log\left( {\frac{{2\sqrt {1 \times 2} }}{{1 + 2}}} \right)^{{\left( {\frac{{2\sqrt {1 \times 2} }}{{1 + 2}}} \right)}}$$$$+ (5n)log(\frac{{2\sqrt {1 \times 3} }}{{1 + 3}})^{{(\frac{{2\sqrt {1 \times 3} }}{{1 + 3}})}}$$$$+ (6n)log(\frac{{2\sqrt {1 \times 4} }}{{1 + 4}})^{{(\frac{{2\sqrt {1 \times 4} }}{{1 + 4}})}}$$$$+ (2n + 1)log$$$$\left( {\frac{{2\sqrt {2 \times 2} }}{{2 + 2}}} \right)^{{\left( {\frac{{2\sqrt {2 \times 2} }}{{2 + 2}}} \right)}}$$$$+ (8n - 3)log$$$$\left( {\frac{{2\sqrt {2 \times 3} }}{{2 + 3}}} \right)^{{\left( {\frac{{2\sqrt {2 \times 3} }}{{2 + 3}}} \right)}}$$$$+ (6n)log$$$$\left( {\frac{{2\sqrt {3 \times 3} }}{{3 + 3}})} \right)^{{\left( {\frac{{2\sqrt {3 \times 3} }}{{3 + 3}}} \right)}}$$$$+ (2n)log(\frac{{2\sqrt {3 \times 4} }}{{3 + 4}})^{{(\frac{{2\sqrt {3 \times 4} }}{{3 + 4}})}}$$$$+ (2n)log$$$$\left( {\frac{{2\sqrt {4 \times 4} }}{{4 + 4}}} \right)^{{\left( {\frac{{2\sqrt {4 \times 4} }}{{4 + 4}}} \right)}} ].$$ $$ENT_{GA(G)}=ENT(G) = \frac{(30.027n - 0.996) \log (30.027n - 0.996) + 1.8743n - 0.0045}{30.027n - 0.996}$$.

$$\square$$

**Table 6:** Numerical Comparison of Degree-based Entropies of Polycarbonate for *n* = 1 to 10.

*n*	$ENT_{M_1}$	$ENT_{M_2}$	$ENT_{ReZG_1}$	$ENT_{ReZG_2}$	$ENT_{ReZG_3}$	$ENT_{ABC}$	$ENT_{GA}$
1	3.153	3.249	3.350	3.444	2.779	3.394	3.432
2	3.871	3.970	4.061	4.058	3.609	4.104	4.142
3	4.285	4.384	4.473	4.470	4.058	4.515	4.552
4	4.577	4.677	4.764	4.761	4.367	4.805	4.843
5	4.802	4.902	4.988	4.985	4.604	5.030	5.068
6	4.986	5.087	5.172	5.170	4.794	5.213	5.251
7	5.142	5.242	5.327	5.325	4.955	5.368	5.406
8	5.276	5.376	5.461	5.459	5.093	5.502	5.540
9	5.394	5.495	5.580	5.577	5.214	5.621	5.658
10	5.500	5.601	5.685	5.683	5.322	5.726	5.764

## Logarithmic model

The logarithmic model, commonly employed in linear regression analysis, predicts future outcomes by establishing a logarithmic relationship between an independent variable and a dependent variable. The predictor or explanatory variable, sometimes referred to as the independent variable, is unaffected by changes in other variables (as stated in^34,35^). On the other hand, variations in the independent variable cause changes in the dependent variable. The regression model predicts the dependent variable, also referred to as the result or response variable under analysis. This statistical technique for predictive analysis in data science and machine learning is covered in the articles^36,37^. The line’s equation is given in the article as follows:

$$\begin{aligned} Y=a X +b. \end{aligned}$$

The dependent variable is denoted by *Y*, the independent variable by *X*, the *Y*-intercept by *b*, and the independent variable’s coefficient by *a*.

The logarithmic regression model is a nonlinear regression technique in which the dependent variable follows a logarithmic pattern but has little to no connection with one or more other variables. In this model, the dependent variable is the natural logarithm of the answer. The independent variable, Entropy, is plotted against the independent variable indices as indicated in^38,39^. Simply put, the exponential growth or decay phenomenon is used in a variety of sectors today, including biology, economics, finance, and many others. The logarithmic regression model can be represented as follows:

$$\begin{aligned} Y=a logX +b. \end{aligned}$$

Where *Y* represents the dependent variable, *X* is the independent variable, *ln*(*X*) denotes the natural logarithm of *X*, *b* is the *Y*-intercept, and *a* is the coefficient of the independent variable.

### Logarithmic model for polyester

**Fig. 15:**
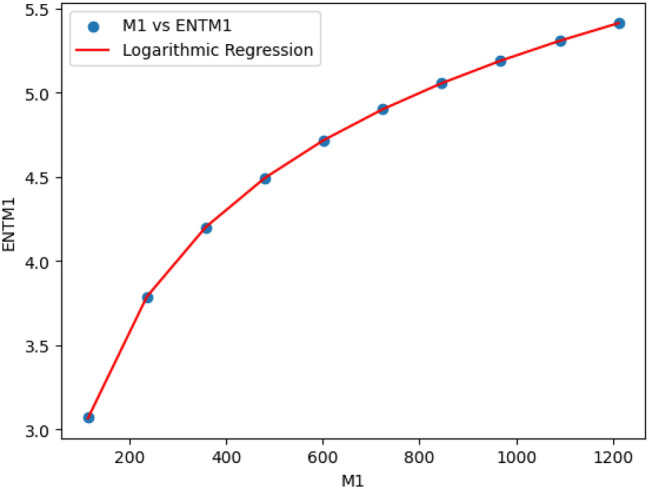
M_1 vs ENTM_1 of polyester.

**Fig. 16:**
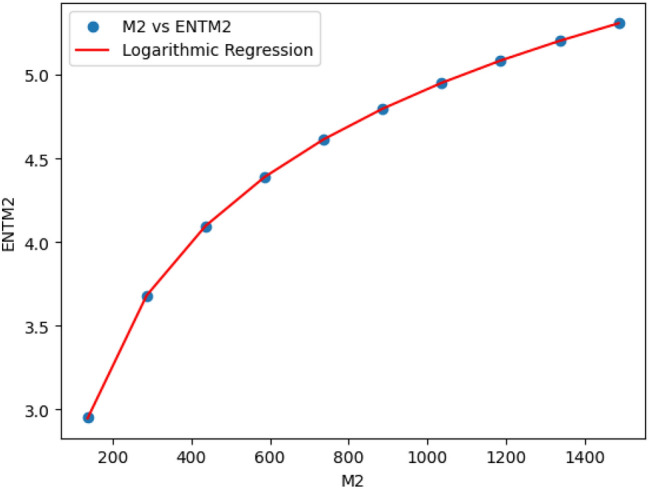
M_2 vs ENTM_2 of polyester.

**Fig. 17:**
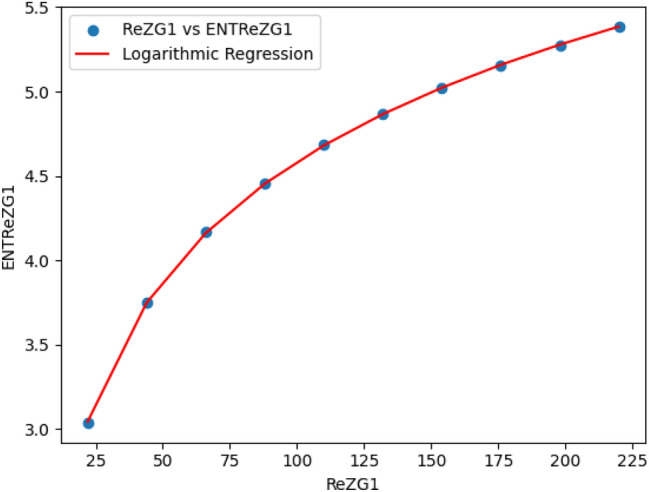
ReZG_1 vs ENTM ReZG_1 of polyester.

**Fig. 18:**
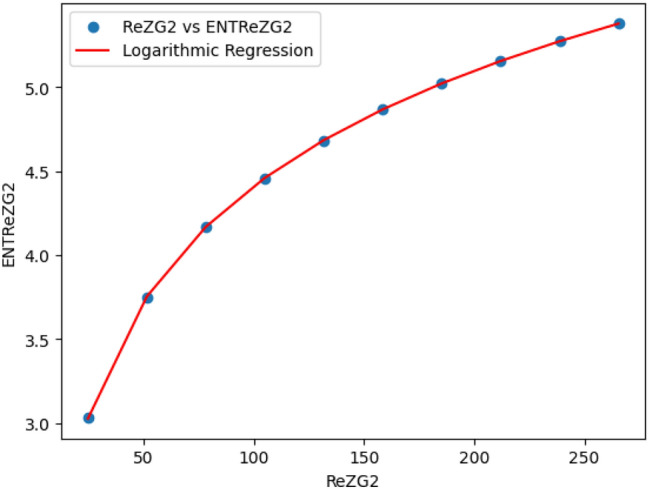
$ReZG_2$ vs $ENT ReZG_2$ of Polyester.

**Fig. 19:**
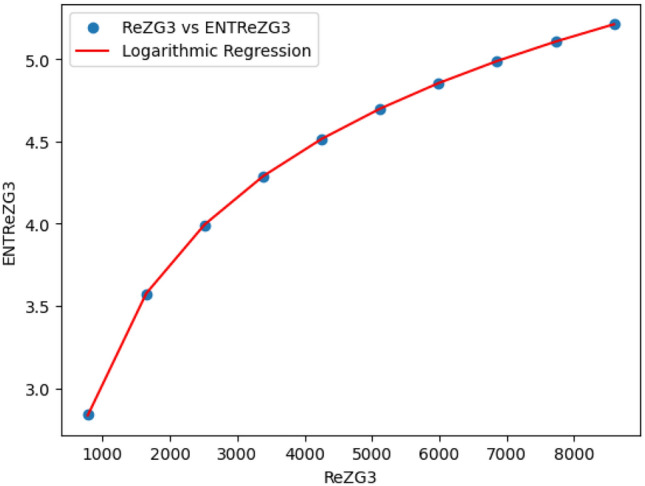
$ReZG_3$ vs $ENT ReZG_3$ of Polyester.

**Fig. 20:**
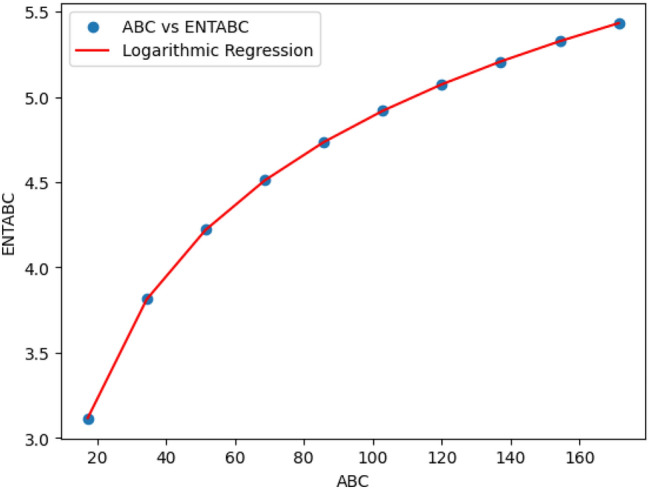
*ABC* vs *ENTABC* of polyester.

**Fig. 21:**
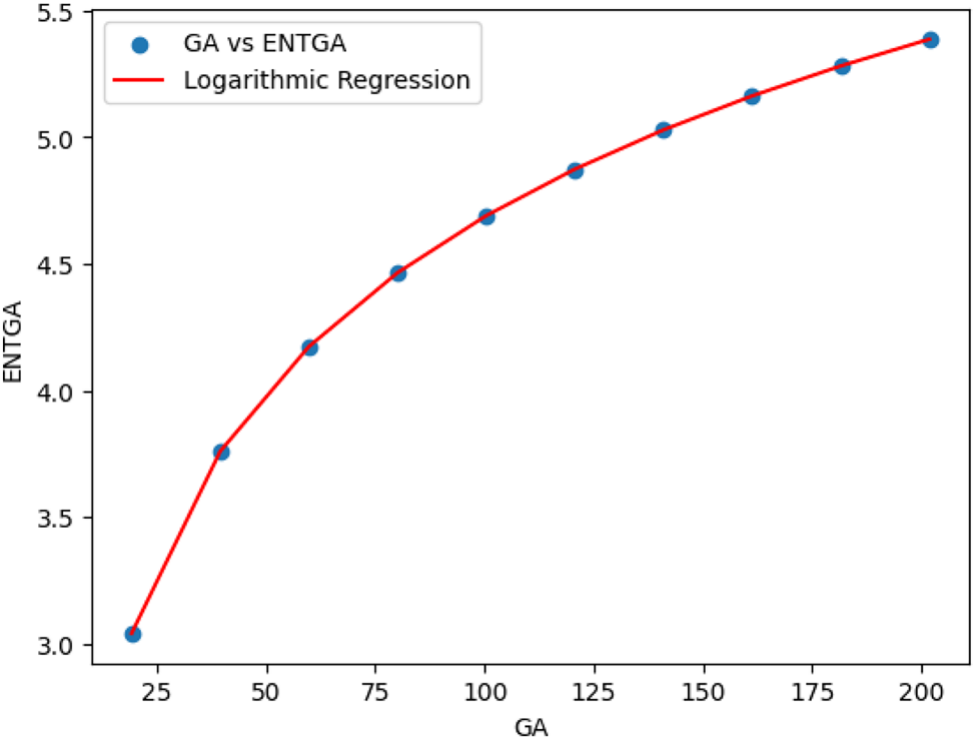
*GA* vs *ENTGA* of polyester.

The statistical values for logarithmic model of polyester are derived from Tables 2 and 3. The Table 7 and Figs. 15,16,17,18,19,20 and 21 shows the statistical values for logarithmic models that analyze the relationship between Entropy and indices for polyester. These models have very high correlation coefficients (R) and coefficients of determination $(R^2)$, with values near 1, which means that they explain over 99.9% of the variability in Entropy. The standard errors $(S_E)$ are very low, ranging from 0.0007 to 0.001, indicating precise predictions. Additionally, the F-statistics are extremely high, and the p-values are below 0.05, providing strong evidence of the models’ significance. These results show that the logarithmic models are highly effective and reliable in understanding the relationship between the indices and the Entropy of polyester, making them useful for predictions and analysis.

**Table 7:** The Statistical Values Using Logarithmic Model for Connecting Units of Polyester.

$Logarithmic \ Model$	$R$	$R^2$	$S_E$	$F$	$P-value$
$ENT_{M_1}=0.9931ln[M_1]-1.6369$	0.9999	0.9999	0.002	1243491.042	4.684E-22
$ENT_{M_2}=0.9877ln[M_2]-1.7058$	0.9999	0.9999	0.003	445794.7911	2.835E-20
$ENT_{ReZG_1}=1.0162ln[ReZG_1]-0.0960$	0.9999	0.9999	0.004	299577.556	1.390E-19
$ENT_{ReZG_2}=0.9886ln[ReZG_2]-0.1407$	0.9999	0.9999	0.003	485590.922	2.014E-20
$ENT_{ReZG_3}=0.9900ln[ReZG_3]-3.7565$	0.9999	0.9999	0.002	6709494.075	5.526E-21
$ENT_{ABC}= 0.2536ln[ABC]+1.0067$	0.9999	0.9999	0.001	1344208.171	3.430E-22
$ENT_{GA}=0.9975ln[GA]+0.0921$	0.9999	0.9999	0.0007	9693762.702	1.268E-25

### Logarithmic model for polycarbonate

**Fig. 22:**
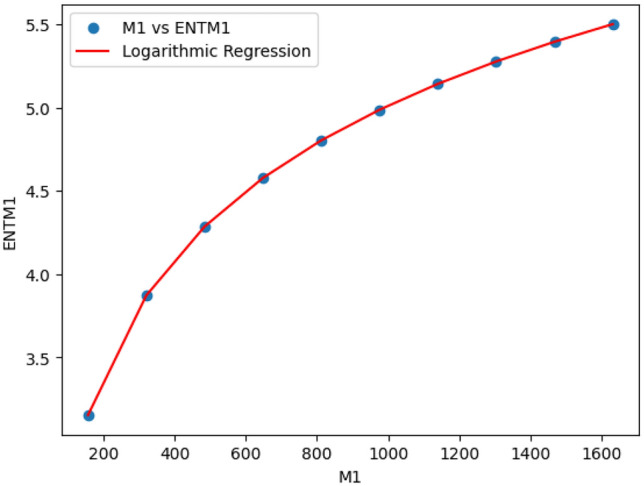
$M_1$ vs $ENTM_1$ of polycarbonate.

**Fig. 23:**
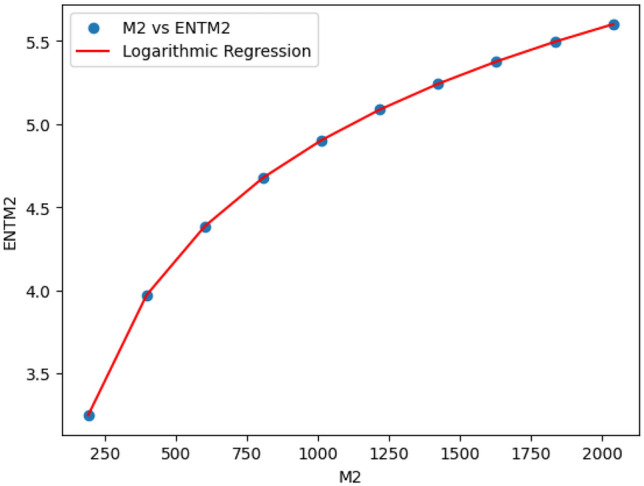
$M_2$ vs $ENTM_2$ of polycarbonate.

**Fig. 24:**
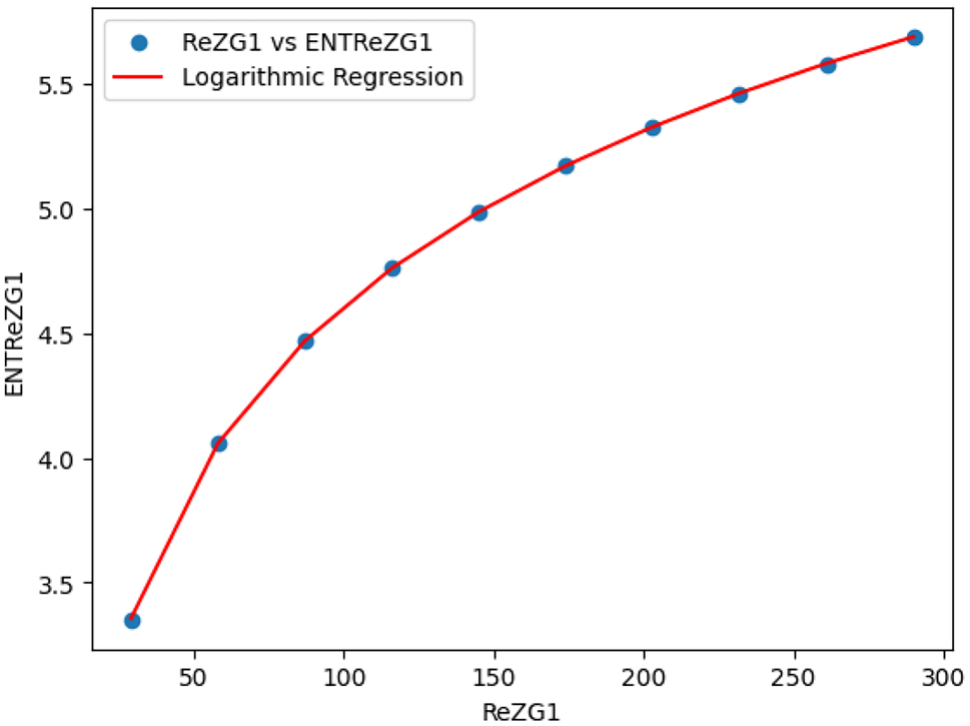
$ReZG_1$ vs $ENT ReZG_1$ of polycarbonate.

**Fig. 25:**
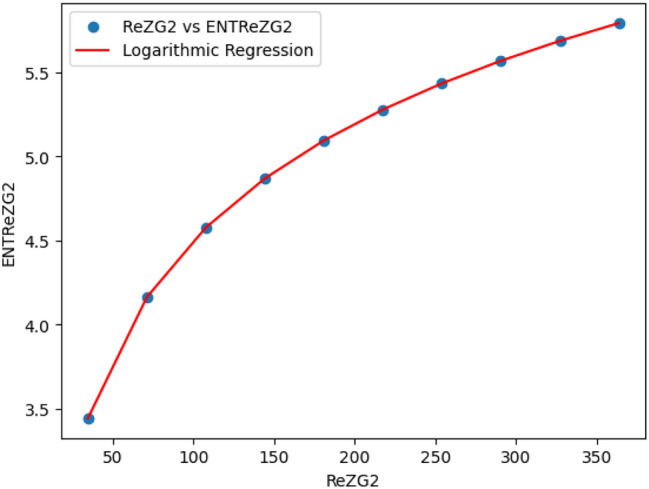
$ReZG_2$ vs $ENT ReZG_2$ of polycarbonate.

**Fig. 26:**
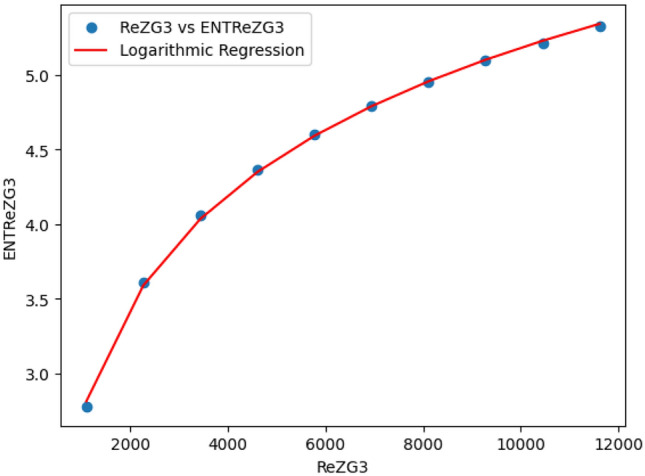
$ReZG_3$ vs $ENT ReZG_3$ of polycarbonate.

**Fig. 27:**
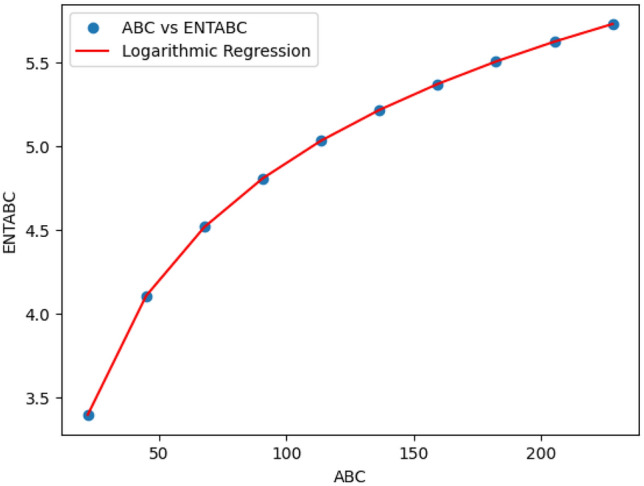
*ABC* vs *ENTABC* of polycarbonate.

**Fig. 28:**
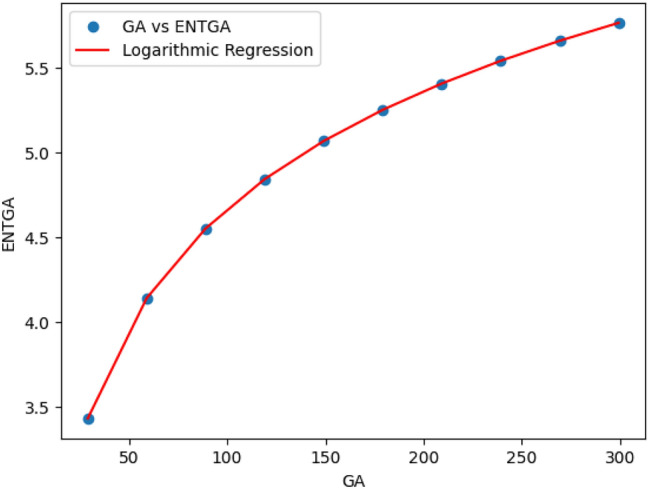
*GA* vs *ENTGA* of polycarbonate.

The statistical values for logarithmic model of polycarbonate are derived from Tables 5 and 6. The Table 8 and Figs. 22,23,24,25,26,27 and 28 shows statistical values for logarithmic models that analyze the relationship between Entropy and indices for polycarbonate, emphasizing their outstanding performance. All models display extremely high correlation coefficients ($R=0.999$) and coefficients of determination ($R^2$ close to 1), indicating that over 99.9% of the variability in Entropy is explained by these models. The standard errors ($S_E$) are exceptionally low, ranging from 0.0002 to 0.020, reflecting high precision in predictions. The F-statistics are significantly large, further affirming the robustness of the models, while the p-values are notably small ($<0.05$), demonstrating the statistical significance of all relationships. These results confirm the effectiveness, precision, and reliability of the logarithmic models in capturing the relationship between Entropy and the indices for polycarbonate, making them valuable tools for predictive and analytical purposes.

**Table 8:** Statistical analysis of the logarithmic model for connectivity units of polycarbonate.

Logarithmic model	$R$	$R^2$	$S_E$	$F$	$P-value$
$ENT_{M_1}=0.9998ln[M_1] -1.8959$	0.999	0.999	0.0002	58301679.590	9.693E-29
$ENT_{M_2}=0.9980ln[M_2]-2.0041$	0.9999	0.9999	0.0006	12140884.549	5.154E-26
$ENT_{ReZG_1}=1.0130ln[ReZG_1]-0.0551$	0.9999	0.9999	0.003	414071.096	3.809E-20
$ENT_{ReZG_2}=0.9992ln[ReZG_2]-0.1006$	0.9999	0.9999	0.001	3489156.262	7.556E-24
$ENT_{ReZG_3}=1.0724ln[ReZG_3]-4.6958$	0.9997	0.9994	0.020	14504.340	2.525E-14
$ENT_{ABC}=1.0004ln[ABC]+0.2949$	0.9999	0.9999	0.0003	41206416.161	3.884E-28
$ENT_{GA}=0.651ln[GA]+0.099$	0.9999	0.9999	0.0002	62921885.127	7.145E-29

## Comparison and discussion

The comparison between polyester and polycarbonate in this study revolves around their structural complexity as analyzed through degree-based topological indices and Entropy measures. Polycarbonate consistently exhibits higher values for all computed indices, such as the Zagreb indices, redefined Zagreb indices, atom bond connectivity index, and geometric arithmetic index. These results reflect polycarbonate’s denser and more intricate molecular framework compared to polyester. Similarly, Entropy measures are also higher for polycarbonate, highlighting its greater degree of randomness and structural disorder.

Logarithmic regression models demonstrate strong correlations between the indices and Entropy measures for both materials, with R² values near unity, affirming the effectiveness of these mathematical tools in modeling polymer behavior. However, polyester’s simpler structure results in slightly more consistent statistical predictions, evidenced by lower standard errors in its logarithmic models.

From an application perspective, polyester’s straightforward molecular design aligns with its widespread use in textiles, packaging, and daily-use items, while polycarbonate’s robustness and stability make it ideal for engineering and high-impact applications like automotive parts, construction materials, and safety equipment. This duality underscores the diverse utility of these polymers, guided by their structural properties and topological characteristics.

## Conclusion

The study effectively demonstrates the utility of graph-theoretical tools, such as degree-based topological indices and Entropy measures, in analyzing the molecular structures of polyester and polycarbonate. By comparing these indices and Entropies, it highlights polycarbonate’s more complex and robust structure, characterized by higher connectivity, randomness, and stability, making it suitable for high-performance and impact-resistant applications. In contrast, polyester’s simpler molecular framework aligns with its versatility and cost-effectiveness for everyday use. The strong correlation between the indices and Entropy measures, observed through logarithmic regression models, underscores the reliability of these metrics in predicting polymer properties. This provides valuable insights for material design and engineering advancements.

For future research, integrating environmental impact assessments and exploring other polymers using similar methodologies could broaden the scope and applicability of these findings.

## Data Availability

The data sets used and/or analysed during the current study available from the corresponding author on reasonable request.
